# T-ALL Cells as Tool Cells for CAR T Therapy

**DOI:** 10.3390/vaccines11040854

**Published:** 2023-04-17

**Authors:** Anqi Ren, Yuan Zhao, Haichuan Zhu

**Affiliations:** Institute of Biology and Medicine, College of Life and Health Sciences, Wuhan University of Science and Technology, Wuhan 430081, China

**Keywords:** immunotherapies, T-cell acute lymphoblastic leukemia, CAR T, CAR Jurkat

## Abstract

T-cell acute lymphoblastic leukemia (T-ALL) is a hematologic malignancy derived from T cells. Numerous CAR T therapies have been successfully applied to treat hematologic malignancies in the clinic. Nevertheless, there remain several challenges to the extensive application of CAR T cell therapy in T cell malignancies, especially in T-ALL. The main reason for CAR T therapy limitations is that T-ALL cells and normal T cells share antigens, which improves the difficulty of sorting pure T cells, resulting in product contamination, and would lead to CAR T cell fratricide. Thus, we considered creating a CAR on T-ALL tumor cells (CAR T-ALL) to prevent fratricide and eliminate tumor cells. We found that T-ALL cells transduced with CAR would actually commit fratricide. However, CAR T-ALL could kill only tumor cells on T-ALL cell lines, and other types of tumor cells had no killing function after being transferred with CAR. Furthermore, we created CD99 CAR with expression controlled by the Tet-On system on Jurkat cells, which could avoid the fratricide of CAR T-ALL during proliferation, ensuring the controllability of the killing time and effect. Jurkat transduced with a CAR-targeting antigen, which was expressed on other cancer cells, could kill other cancer cell lines, demonstrating that T-ALL cells could be used as tool cells for cancer therapy. Our study supplied a new feasible treatment regimen for cancer treatment in the clinic.

## 1. Introduction

T-cell acute lymphoblastic leukemia is a hematologic tumor derived from T cells, which develop rapidly and have a high recurrence rate after therapy; T-cell acute lymphoblastic leukemia accounts for about 10–15% of acute lymphoblastic leukemia cases in children and 20–25% of that in adults [1,2]. Current treatment protocols make for an average survival rate of 70% for T-ALL patients; however, relapse occurs in 20–25% of children and more than half of adult patients, and 50–70% of relapsed patients succumb to disease quickly [3].

Chimeric antigen receptor T-cell (CAR T) therapy has achieved great success in hematologic malignancies, the earliest treating B-cell malignancies [4,5]. However, the issue with CAR T therapy is that it is characterized by poor in vivo persistence due to the exhaustion of T cells, which affects the application of CAR T therapy [6]. Moreover, a common expression of targeting antigens between T-ALL cells and normal T cells would cause several flaws in CAR T therapy, such as fratricide and T cell aplasia [7,8]. CD99 has been demonstrated to have high expression in T-ALL and could be used as a tool for the detection of MRD [9]. Previously, our group identified the 12E7 scFv, a low-affinity CD99 antibody that specifically recognizes leukemia cells over normal blood cells, which could prevent CAR T fratricide. The CAR T cell targeting CD99 has been constructed and proved to have a therapeutic effect on T-ALL [10].

Recently, we found the phenomenon that CD99 CAR-transducing T-ALL cells could cause the tumor cells to kill themselves, and the CAR targeting another antigen expressed on T-ALL cells could result in the same phenomenon. The construction of CD99 CAR included an antigen-binding domain (12E7 scFv), an extracellular CD8 hinge, an intracellular CD28 costimulatory domain, a 4-1BB costimulatory domain, and a CD3 activation domain. Moreover, we also constructed CAR targeting CD7 and CD4, which were expressed on T-ALL cell lines, and CD19, CD30, and Mesothelin (MSLN), which had no expression on T-ALL cell lines. We demonstrated that the phenomenon of fratricide occurred on only T-ALL cells over other tumor cells, which suggested that it could be a strategy to treat T-ALL. Moreover, it also has clinical implications that we do not need to exert great effort to sort complete normal T cells from patients’ blood to produce CAR T cells in the clinical therapy of T-ALL.

## 2. Materials and Methods

### 2.1. Cell Lines and Cell Culture

Jurkat, THP-1, SK-OV-3 cell lines were purchased from the Chinese Academy of Sciences Cell Bank (CASCB, Beijing, China). RDES cell lines were purchased from the American Type Tissue Culture Collection (ATCC, Manassas, VA, USA). Loucy, Raji, and MOLM-13 cell lines were purchased from DSMZ (Brunswick, Germany). CUTLL-1, MOLT-4, OVCAR-8, and KE37 cell lines were kindly provided by Hong Wu Lab at Peking University. The identity of the cells was confirmed by STR loci profiling performed by the aforementioned institutes.

RD-ES cell lines were cultured in DMEM with 10% FBS. Jurkat, MOLT-4, CUTLL-1, KE37, Loucy, THP-1, MOLM-13, Raji, SK-OV-3, and OVCAR-8 cell lines were cultured in RPMI 1640 medium with 10% FBS. The aforementioned cell lines were placed in a 37 °C and 5% CO2 humidified incubator and tested to ensure the lack of mycoplasma contamination.

### 2.2. DNA Constructs and Lentivirus Production

The sequence of 12E7 scFv was derived from patent (number: WO2015161267 A2). The sequence of CD4 scFv was derived from patent (number: WO2007094983 A2) and was optimized. The sequence of CD7 scFv was derived from patent (number: WO03051926 A2). The sequence of CD19 scFv was derived from patent (number: US20140271635 A1). The sequence of CD30 scFv was derived from patent (number: CN106589139 A). The sequence of MSLN scFv was derived from patent (number: WO9928471 A2). The CAR backbone is composed of a CD8 hinge, an intracellular CD28 costimulatory domain, a 4-1BB costimulatory domain, and a CD3 activation domain. To produce CD99, CD4, or CD7 CAR lentivirus, 293 T cells were transfected with a combination of plasmids containing PTK-CAR, pMDLg-pRRE, pRSV-rev, and pMD 2.G.

### 2.3. Flow Cytometry

Flow cytometry was performed by using a Beckman CytoFLEX flow cytometer. Cell lines were incubated on ice for 30 min in the dark with antibodies purchased from BioLegend at the recommended dilution before being washed three times with PBS containing 2% FBS (Gibco, Grand Island, NY, USA). Cells were stained with 7-AAD to eliminate dead cells. Positive events were determined for each antibody by using isotype control gating. Strep II antibody (GenScript, Piscataway, NJ, USA, 5A9F9) was used to calculate the efficiency of anti-CD99 CAR transduction. Data analysis was carried out by using FlowJo.

### 2.4. Apoptosis Assays

Before detection, cells were treated with a FITC-Annexin V Apoptosis Detection Kit (Yeasen Biotech, Shanghai, China). The FITC-Annexin V and propidium iodide were used for dual staining in accordance with the instructions; then, the cells were analyzed with a CytoFLEX flow cytometer (Beckman Coulter, Brea, CA, USA). We distinguished between living, dead, early apoptotic, and apoptotic cells. The target of our comparison was the relative proportion of early apoptotic cells and apoptotic cells.

## 3. Results

### 3.1. T-ALL Cell Lines Transduced with CAR Has Similar Cytotoxicity to That of CAR T Cells

**Figure 1:**
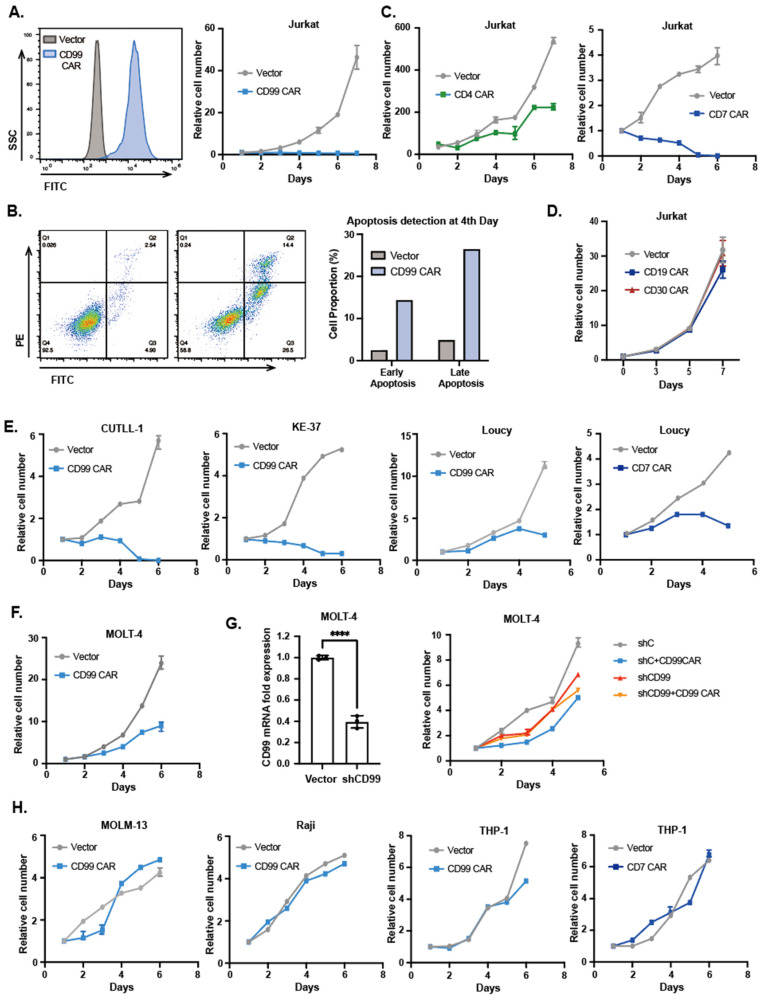
Cell proliferation and transfection efficiency of Jurkat cell lines after transduction with CD99. The cell count was performed at 24 h after transduction and once a day. The transfection efficiency was assessed at 48 h after transduction and once every two days (**A**). Apoptosis assay detected via Flow cytometry at 96 h after transduction (**B**). Cell proliferation of Jurkat cell lines after transduction with CD4 or CD7. The cell count was performed at 24 h after transduction and once a day (**C**). Relative cell growth of Jurkat cell lines with CD19 CAR, or CD30 CAR. The cell count was performed at 24 h after transduction and once every two days (**D**). Cell proliferation of other T-ALL cell lines after transduction with CD99 or CD7 (**E**). Cell proliferation of MOLT-4 cell lines (**F**) and CD99 knock-down stabled MOLT-4 cell lines (**G**, **right**) after transduction with CD99; RT-qPCR detected knock-down efficiency (**G**, **left**). Data are mean ± SD (Two-tailed unpaired Student’s t test, **** *p* < 0.0001). Cell proliferation of non-T-ALL cell lines after transduction with CD99 or CD7 (**H**).

Because T-ALL cells are derived from normal T cells, they express nearly the same antigens as normal T cells, such as CD5 or CD7 [11,12]. This shared antigen expression can cause CAR T cells to fratricide, limiting CAR T cell proliferation in vitro. We discovered an intriguing phenomenon in which the CD99 CAR-transduced Jurkat cell line, a T-ALL cell line, started fratricide similarly. The transfection efficiency of CD99 CAR attained almost a hundred percent and did not decrease by a large margin, within a week, as time went on (Figure 1A). Moreover, the anti-CD99 CAR Jurkat cells showed robust cytotoxicity, inhibiting growth and causing final cell death. To investigate the mechanism of T-ALL cell death after the transduction of CAR, we adopted an Annexin V/propidium iodide apoptosis assay to detect the death of CAR Jurkat cells, with results showing that CAR Jurkat cells underwent mass mortality after 96 h (Figure 1B). Thus, we supposed that apoptosis is the main cause of CAR Jurkat cell lines.

Moreover, we transduced CAR targeting another antigen, such as CD7 and CD4, which T-ALL cells express, and the CAR Jurkat cells kill themselves in the same way (Figure 1C). CAR Jurkat cells targeting the antigens, CD19 and CD30, which T-ALL cells do not express, had normal proliferation (Figure 1D), proving the specific cell killing of CAR.

Furthermore, to explore whether this phenomenon exists in all T-ALL cells, we transduced CAR on the other T-ALL cell lines, including MOLT-4, CUTLL-1, Loucy, and KE37 cells. After being modified with CD99 or CD7 CAR, these T-ALL cells all blocked growth, leading to cell death (Figure 1E,F). This finding suggested that fratricide was the universal phenomenon in T-ALL cell lines. To verify cell apoptosis due to CD99 CAR T-ALL cells recognizing and attacking CD99^+^ tumor cells, not due to the negative effect of virus transfection, we constructed a CD99-knock-down cell line, which was named shCD99 MOLT-4. CD99 CAR transducing shCD99 MOLT-4 cell lost the attacking target and prolificated normally (Figure 1G), which demonstrated that T-ALL cells still retain the immunocompetence to attack tumor cells, as do normal T cells. 

CD99 has been reportedly associated with several malignancies and has been found to actively contribute to the persistence of tumor malignancies, such as AML, lymphoma, ovarian carcinoma, and Ewing’s sarcoma [13,14,15,16,17]. To explore whether immunocompetence is the specific characteristic of T-ALL cells and whether the phenomenon of CAR tumor cell fratricide exists only on T-ALL cells or on all hematopoietic and non-hematopoietic tumor cells, we selected varieties of tumor cells to construct CD99 CAR tumor cells and tested their proliferation efficiencies, such as THP-1, MOLM-13 cell lines from (human acute myelocytic leukemia, AML), Raji cell line from lymphoma, SKOV-3 cell line from ovarian carcinoma and the RDES cell line from Ewing’s sarcoma. Distinct from T-ALL cells, the other tumor cells did not kill each other and still proliferated normally, although they have high expression of CD99 or CD7, the CAR-targeting antigen (Figure 1H and Figure S1). This finding suggested that only the T-ALL cells, the tumor cells derived from normal T cells, may maintain the immunogenicity that the normal T cells have and could be killed by CAR tumor cells. These findings demonstrated that transducing CAR, which targeted the antigens that the T-ALL cell expressed, on T-ALL cells is a strategy that could be applied for anti-tumor research in T-ALL.

### 3.2. Application of CAR Jurkat Cell Lines Treating T-ALL or Non-T-ALL

**Figure 2:**
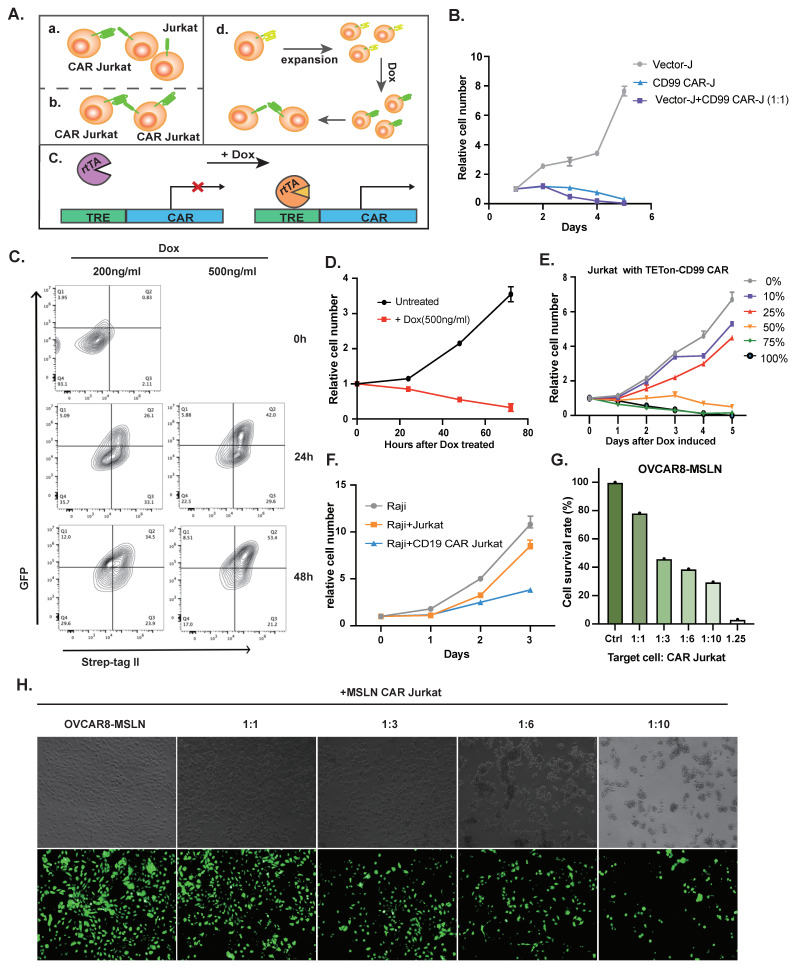
Schematic illustration of CAR Jurkat cells killing untreated (**a**) and CAR-transduced (**b**) Jurkat cell lines; mechanism of the Tet-On system (**c**); mechanism of TET on system (**d**) (**A**). Relative cell growth of Jurkat cell lines mixed with CD99 CAR-transduced Jurkat cell lines (1:1) (**B**). Flow cytometry detect the expression of CD99 CAR on Jurkat cell lines at 24 h and 48 h after treatment with Dox (200 ng, 500 ng) (**C**). Relative cell growth of Jurkat cell lines transduced with Tet-On system after treatment of Dox (500 ng) (**D**). Relative cell growth of Jurkat cell lines mixed with Jurkat cell lines transduced with Tet-On system after treatment with Dox (500 ng) in 5 gradients (10%, 25%, 50%, 75%, 100%) (**E**). Relative cell growth of Raji cell lines mixed with untreated Jurkat cell lines or CD99 CAR-transduced Jurkat cell lines (1:3) (**F**). Relative cell growth of MSLN-overexpressed OVCAR-8 cell lines mixed with MSLN CAR-transducing Jurkat cell lines in different proportions (**G**). MSLN-overexpressed OVCAR-8 cell lines has the expression of green fluorescent protein (GFP); the survival of OVCAR-8 cell lines after co-incubation with MSLN CAR Jurkat cell lines after 48 h was visualized via fluorescence microscopy (**H**).

TET-on system is a controlled expression system in which the target gene undergoes transcription via doxycycline (Dox) induction. The rTRA, a fusion protein of rTetR and VP16, is the opposite phenotype of tTA and could not bind to TRE. During dox induction, the conformation of rtTA is altered, binding to TRE and turning on gene expression [18] (Figure 2A).

To exclude the possibility that CD99 CAR blocks the CD99 expression on T-ALL cells and affects the survival of T-ALL cells, we mixed the CD99 CAR-J cells and untreated Jurkat cells in the same proportion. Both the CAR-J cells and untreated Jurkat cells finally died, which proved that the CAR-J cells recognized the CD99 rather than blocking the expression of CD99 and finally caused Jurkat cell death (Figure 2B). 

To prevent the CD99 CAR-J cells from causing fratricide before being applied to kill other untreated T-ALL cells, we adopted the Tet-On system to render the expression of the CD99 CAR controllable. CD99 CAR is expressed only on the Jurkat cells after Dox induction. To determine the optimal concentration, we designed two concentration gradients of Dox, 200 ng/mL and 500 ng/mL, and detected their induction efficiency via flow cytometry (FCM). FCM revealed that Dox-induced cells expressed CD99 CAR, and the 500 ng/mL Dox-inducible efficiency was higher than the 200 ng/mL Dox-inducible efficiency, so we finally chose 500 ng/mL to be the working concentration of Dox (Figure 2C). We ascertained the killing efficiency of CD99 CAR-J cells with the Tet-On system (Figure 2D). We also noted the proliferation of T-ALL cells mixed in different proportions of Dox-induced cells and obtained the result that more than half of the proportion of Dox-induced cells may kill complete T-ALL cells (Figure 2E). Since the transfection efficiency of the TET-on system and induction efficiency of Dox were not one hundred percent, we speculated that a lower proportion of Dox-induced cells could successfully lead to complete T-ALL cell death.

Moreover, we constructed CD19 or MSLN CAR Jurkat cell lines, which could proliferate normally and have cytotoxicity to Raji cell lines and MSLN-overexpressed OVCAR-8 cell lines (Figure 2F–H). This revealed that T-ALL cell lines could be the tool cells to treat other cancers.

## 4. Discussion

CAR T therapy has achieved significant success in B-cell malignancies [4]. T-ALL shows different kinetic patterns of disease response from B lymphoblastic leukemia (B-ALL) [2,19]. Fratricide, which is caused by the same antigen expression both on malignant T cells and normal T cells, is the main challenge during CAR T therapy. Moreover, CAR T cells would also kill normal T cells, leading to T cell aplasia. It is still difficult to adapt CAR T immunotherapy in T cell malignancies, especially in T-ALL, because of lacking tumor-specific antigens in malignant T cells and product contamination [7].

CD99 is a potential targeting antigen for CAR T immunotherapy of CD99^+^ leukemia cells, preventing the development of several hematologic malignancies [10]. The 12E7 scFv, a low-affinity CD99 antibody that will not specifically recognize normal blood cells, could avoid CAR T cells fratricide. Undisputed, CD99 CAR T therapy could be an efficient approach to treating T-ALL.

Our group tried to construct CD99 CAR on Jurkat cells, a CD99 high expressing T-ALL cell line, and surprisingly found that the CD99 CAR Jurkat cells stopped growth and killed themselves. In a further study, we constructed CD99 CAR on another T-ALL cell line and identified that all these CD99 CAR T-ALL cells caused fratricide and complete death. CD4 or CD7 CAR T-ALL cells maintained fratricide, verifying the cytotoxicity of CAR targeting specific antigens. Furthermore, this phenomenon did not exist in other hematologic malignancy cells or solid tumor cells, which demonstrated that T-ALL cells derived from mutational T cells may reserve T cell-specific immunocompetence. CAR Jurkat cells targeting antigens that were not expressed on T-ALL and CD99-knock-down MOLT-4 cells lost the cytotoxicity but transduced CD99 CAR, showing the targeting specificity of CD99 CAR.

In addition, we constructed the Tet-On system, which could control the expression of CD99 CAR via Dox induction. Jurkat, which was transduced with the Tet-On system, successfully expressed CD99 CAR after Dox treatment. Since the CD99 CAR Jurkat cells kill themselves and untreated cells at the same time, too few proportions of CD99 CAR Jurkat may be depleted before they completely kill the untreated cells. In our research, more than half of TET-on stable expressing Jurkat cells could result in complete cell death before killing themselves. Since only about 50 percent of Jurkat cells with the TET-on system became CD99 CAR-expressing after Dox induction, the proportion of Jurkat cells with the TET-on system could be much less in fact. Jurkat cell lines transduced with the CAR-targeting antigen, which was expressed on Raji or OVCAR-8 cell lines, could inhibit the cell growth of Raji and OVCAR-8 cell lines, demonstrating that T-ALL cell lines could be used as tool cells for both T-ALL and non-T-ALL treatment. NK-92 cell lines from a patient with non-Hodgkin lymphoma have been used for CAR T therapy and achieved great results. CD19-CAR NK-92 cells were created and were proven to specifically lyse CD19-expressing B-precursor leukemia cell lines and lymphoblasts from leukemia patients [20]. What is more, the clinical safety and anti-tumor activity of CD33-CAR NK-92 has been tested in relapsed and refractory AML patients [21]. Radiation was used to treat NK-92 cells before clinical application, ensuring the safety and sufficient cytotoxicity of NK-92 cells [22]. The application of NK-96 cells led us to believe that T-ALL cell lines had the potential as tool cells for CAR T therapy in the clinic. Nonetheless, most of the CAR constructs that were used to generate CAR NK-92 were designed optimally for CAR T cells rather than NK cells, such as the costimulatory molecule CD28, which has no expression in NK cells, and may reduce the cell viability of NK-92 [23]. Based on the clinical data, CAR-NK therapy was slightly less effective than CAR T therapy [24]. Our research provided a fresh cell strategy for CAR T therapy of cancer treatment. If T-ALL cell lines are regarded as tool cells that may develop into new cancers in vivo, irradiation may be a good strategy. Moreover, we supplied a notion of treating T-ALL by transducing CAR in vivo, in which the CAR T-ALL cell lines could not proliferate and have no risk of cancer development. 

## 5. Conclusions

Collectively, we found that CAR T-ALL cells could commit fratricide. This phenomenon could solve the problem of CAR T cells product contamination, which sorted T cells mixed with tumor cells and led to a significant adverse consequence. Thus, it could be a promising strategy to prevent the progression of T-ALL. Moreover, this phenomenon exists only in T-ALL cells, from which we speculate that reserve T cell-specific immunocompetence exists. CAR Jurkat had proven cytotoxicity to other non-T-ALL cell lines which have the CAR-targeting antigen, demonstrating its utilization potentiality as a tool cell for cancer treatment. Although we had proved the CAR Jurkat therapeutic regimen in vitro, further experiments still need to be carried out in vivo. A novel approach, in which human CAR T cells are generated by injecting the Adeno-associated virus (AAV) vector with the CAR gene, has been applied in vivo [25,26]. Thus, we believe that our study substantially simplifies the manufacturing process of CAR T cells and has application value in the clinic. 

## Data Availability

Not applicable.

## Supplementary Materials

The following supporting information can be downloaded at: https://www.mdpi.com/article/10.3390/vaccines11040854/s1, Figure S1: Cell proliferation of non-T-ALL (RDES, SK-OV-3) cell lines after transduced with CD99.
