# Supplementary figures and images for: T-ALL Cells as Tool Cells for CAR T Therapy
Source: Vaccines (Basel). 2023 Apr 17;11(4):854. doi: 10.3390/vaccines11040854 (PMC10142624; doi:10.3390/vaccines11040854)

Figure S1: “Fratricide” is not triggered at Non T-ALL cells.

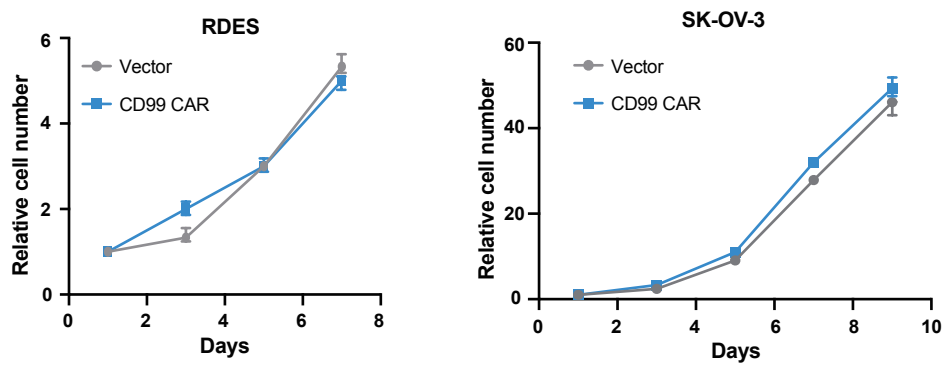

Supplement: Supplementary file 1 [file vaccines-11-00854-s001.zip › vaccines-2285025-supplementary.pdf]
